# Therapeutic Use of Virtual Reality for Patients With Fibromyalgia and Chronic Neck Pain: Randomized Controlled Trial

**DOI:** 10.2196/81158

**Published:** 2026-01-23

**Authors:** Edurne Úbeda-D’Ocasar, Yaiza Moreno-Crespo, Eduardo Cimadevilla-Fernández-Pola, Juan Hernández-Lougedo, Álvaro Navas-Mosqueda, Mario Caballero-Corella, Noemí Mayoral-Gonzalo, Blanca Pedauyé-Rueda, María Jesús Fernández-Aceñero, Juan Pablo Hervás-Pérez, Cristina Ojedo-Martín

**Affiliations:** 1 Physiotherapy and Health Research Group (FYSA), Faculty of Health Sciences – HM Hospitals, Camilo José Cela University, Villanueva de la Cañada, Madrid, 28692, Spain; 2 Instituto de Investigación Sanitaria HM Hospitales, Madrid, Spain; 3 Department of Legal Medicine, Psychiatry and Pathology, Facultad de Medicina, Universidad Complutense de Madrid, Fundación de Investigación Clínico San Carlos (IDiSCC), Madrid, Spain; 4 Department of Chemistry in Pharmaceutical Sciences, Analytical Chemistry Unit, Faculty of Pharmacy, Complutense University of Madrid, Plaza Ramón y Cajal s/n, Ciudad Universitaria, Madrid, Spain, +34 918153131

**Keywords:** chronic neck pain, exercise, fibromyalgia, virtual reality, randomized controlled trial

## Abstract

**Background:**

Fibromyalgia (FM) causes widespread pain, fatigue, and cognitive abnormalities. Cervical pain is a common and debilitating symptom.

**Objective:**

This study aims to evaluate the effectiveness of virtual reality (VR) as a treatment for chronic cervical pain experienced by patients with FM.

**Methods:**

A single-blind randomized clinical trial was conducted. A total of 56 women were randomly assigned to 3 groups: G1 (VR+cervical mobility exercises), G2 (cervical mobility exercises), and the control group. Therapy was administered twice a week for 4 weeks. Variables such as disease impact, quality of life, kinesiophobia, pain, range of motion, fatigue, and treatment adherence were measured.

**Results:**

The mean age of the participants was 54.26 (SD 7.7) years. Participants were overweight, with a mean BMI of 28.7 (SD 7.8). The mean visual analog scale value was 6.72 (SD 1.8). The baseline values for age, BMI, visual analog scale, algometric measures, and functional capacity (measured using the Timed Up and Go test, cervical rotation, and lateral displacement) were similar across the 3 groups. Following the intervention therapy, the control group did not exhibit notable improvement (mean 3.5, SD 1.4; differences of mean values –0.46, 95% CI –1.1 to 0.2; *P*=.15), particularly in pain perception, while both therapy groups did show improvements (G1: mean 3.8, SD 1.1; differences of mean values 1.2, 95% CI 0.78-1.54; *P*<.001; G2: mean 2.8, SD 1.8; differences of mean values 1.2, 95% CI 0.66-1.7; *P*<.001). Both intervention groups improved significantly compared to control postintervention in FM impact (CG vs G1: differences of mean values 9.31, 95% CI 14.7-3.8; *P*<.001; CG vs G2: differences of mean values 8.4, 95% CI 13.84-3.06; *P*<.001), central sensitization (CG vs G2: differences of mean values 7.53, 95% CI 12.12-2.95; *P*<.001), and cervical disability (CG vs G2: differences of mean values 6.44, 95% CI 9.93-2.94; *P*<.001). However, at 1 month, only G1 maintained superior improvements across all measures, including a reduction in kinesiophobia (G2: differences of mean values 6.2, 95% CI 4.7-9.8; *P*<.001), indicating a more sustained effect of the combined approach.

**Conclusions:**

The combination of VR with cervical mobility and strengthening exercises produced superior and sustained improvements in women with FM compared to exercise alone or control. Significant benefits were observed in disease impact, central sensitization, cervical disability, and kinesiophobia, with effects maintained at 1 month only in the VR group. These findings support VR as an effective adjunct to enhance symptom management and treatment adherence in FM.

## Introduction

Fibromyalgia (FM) is a disease that causes widespread chronic pain and intense fatigue along with many other symptoms [1-3]. Historically, there was some skepticism among health care professionals regarding the existence of this disease and its classification as a mental disorder. Such skepticism may have arisen from concerns about the optimal use of resources in providing care for individuals with FM [3,4]. It is estimated that this disease affects between 2% and 4% of the Spanish population, making it the most common cause of diffuse chronic musculoskeletal pain. Notably, the prevalence is much higher in women, with a ratio of 8:1 [5-7]. Its incidence is increasing because of the improvements in diagnostic criteria and advances in research that are bringing this pathology to light [7,8]. Given its high prevalence, this disease represents a significant public health concern [9,10].

Currently, there are no specific diagnosis tests available for confirming an FM diagnosis. Historically, an FM diagnosis was based on the presence of widespread pain in at least 11 of 18 designated tender points for a duration of 3 months. However, this criterion proved insufficient to capture the complexity of the disorder. Consequently, more comprehensive diagnostic frameworks have been developed, including a range of associated symptoms such as fatigue, joint stiffness, cervical and lumbar pain, muscle weakness, depressive symptoms, gastrointestinal issues, cognitive impairments, and balance alterations. Collectively, these manifestations significantly compromise the quality of life of individuals affected by FM. This pain can become disabling during flare-ups and even become chronic, often being a cause of recurrent absences from work [11].

Unlike pain associated with a sedentary lifestyle or poor posture, neck pain in FM results from a complex interplay between physiological, neurological, and psychological factors. Chronic pain often involves the cervical spine, leading to acute and prolonged pain episodes, along with muscle tension in the suboccipital, trapezius, and elevator scapulae muscles. It is thought that several factors may contribute to this chronic pain, including central pain sensitization, nervous system dysregulation, sleep disturbances, musculoskeletal changes, and psychological and emotional factors [11-13].

In recent years, new therapeutic approaches have been explored to improve symptomatology and thus patients’ quality of life. One of the main challenges in treating patients with chronic pain, particularly those with FM, is their low adherence to physical therapy exercises. This may be due to factors such as severe pain and fatigue, a fear of worsening symptoms, a lack of understanding about exercise benefits, inadequate support and supervision, and frustration with slow improvement. To address these issues, it would be beneficial to adopt a comprehensive approach that involves educating patients regarding the benefits of exercise, customizing programs according to individual needs, providing ongoing support via health care professionals, managing pain and fatigue during exercise, setting realistic expectations, and recognizing achievements [14-16]. It may be beneficial to consider a treatment plan that incorporates aerobic activities, strength training, and stretching exercises [17-19]. However, this type of therapy may be limited by kinesiophobia. One possible solution to this issue could be the use of virtual reality (VR) technology. Exercise interventions delivered through VR have shown superior efficacy in alleviating the core symptoms of FM, including pain, fatigue, and stiffness, while also promoting improvements in balance and postural control [20]. The affordability of portable VR devices, combined with their sustained effectiveness as a nonpharmacological intervention for chronic pain management, positions VR as a promising tool with potential future applications as an analgesic modality [21]. Numerous randomized controlled trials have demonstrated that VR constitutes an alternative and accessible therapeutic approach for pain management [22].

Immersive VR is an innovative and interactive technology that generates 3D scenarios, enabling patients to experience highly realistic simulations while interacting with the virtual environment using their own hands. VR has been researched and applied in various contexts for pain management, demonstrating considerable potential as a therapeutic tool. The effectiveness of this therapeutic approach has been demonstrated for patients with chronic pain, as it addresses several factors, such as fatigue, kinesiophobia, and range of motion (ROM) [23]. This therapy can be adapted to suit the specific requirements of each patient, providing the option to adjust the level of difficulty from low to intermediate or high. Additionally, it offers a variety of game modes that could be beneficial in targeting different clinical conditions, such as low back pain, neck pain, and balance disorders [24,25].

The therapeutic mechanisms and components of VR are distraction, activity management and behavioral activation, skills-based cognitive behavioral therapy, relaxation training and biofeedback, positive emotion induction, neuromodulation, physical rehabilitation, and reduction of kinesiophobia [8,23,26].

VR can be categorized into 2 main types: immersive and nonimmersive. Immersive VR involves the use of head-mounted displays or VR goggles that fully cover the visual field and may include motion sensors for the hands or feet. In contrast, nonimmersive VR is accessed through conventional screens or computers, without any device that isolates the user from the external environment [27] .

Chronic pain has been extensively investigated over the past decades, and research efforts persist in identifying novel strategies to mitigate its impact and enhance patients’ quality of life. VR in both modalities has demonstrated efficacy in reducing chronic musculoskeletal pain. These findings support the integration of VR as a therapeutic tool in clinical populations affected by conditions associated with this type of pain [28].

Patients may perceive amplified motion in the virtual world, depending on the configuration of the headset used. This could result in small neck flexion in real life being rewarded with greater apparent motion in VR, which might increase kinesiophobia [26]. Studies have indicated that for patients with chronic low-back pain, kinesiophobia may be addressed through VR interventions [20,29,30]. VR helps to distract an individual from painful stimuli while exercising in a way that improves their perception of pain [31]. In addition, there have been indications that improvements in fatigue, sleep quality, and ROM may also be achieved [9].

In this study, we aimed to evaluate the effectiveness of VR as a therapeutic option for neck pain in patients with FM. We hypothesized that VR could decrease cervical pain and kinesiophobia and increase ROM, facilitating improvements in quality of life and adherence to treatment.

## Methods

### Trial Design

A single-blind, randomized experimental clinical trial was conducted among women diagnosed with FM recruited through the Association of Fibromyalgia, “AFINSFACRO,” in Móstoles, Spain. Following the selection of patients who met the inclusion criteria and the collection of informed consent forms and information sheets, according to ethical standards, the patients were randomly assigned to 1 of the 3 groups. The first group performed 20 minutes of exercise plus 10 minutes of VR (G1), the second group performed 30 minutes of exercise (G2), and the control group (CG) did not perform any exercise or VR treatment.

This study was registered at ClinicalTrials.gov (NCT05933941) and approved by the Clinical Research Committee of the Hospital Clínico San Carlos (Madrid, Spain; ID: VRTCNPPFM-13/07/2023). The research was conducted from April 1, 2024, to January 30, 2025. All participants gave informed consent, and the general scope of this study was explained to them via a participant information sheet. This clinical trial was designed and reported in accordance with the CONSORT (Consolidated Standards of Reporting Trials) guidelines, with specific adherence to the CONSORT-Harms (Consolidated Standards of Reporting Trials Harms Extension) 2022 statement (Checklist 1) to ensure transparent and comprehensive reporting of adverse events and safety outcomes.

### Participants

The QuestionPro survey software (QuestionPro, Inc) was used to calculate the required sample size, considering a 95% CI and a 5% α error. The sample size calculation was based on the study by Gulsen et al [8]. A total of 56 women were enrolled, of whom 2 withdrew for personal reasons unrelated to the study. The inclusion criteria comprised female patients aged 20 to 65 years with a confirmed diagnosis of FM and chronic cervical pain lasting more than 3 months. The exclusion criteria included vertigo, claustrophobia, epilepsy, pregnancy, or refusal to provide informed consent.

### Type of Sampling

A consecutive, nonprobabilistic convenience sampling strategy was applied to participants who met the inclusion criteria.

### Variables and Outcomes

Sociodemographic variables included age, height, weight, BMI, marital status, pain medication use, employment status, smoking history, and comorbid conditions such as restless legs syndrome, chronic fatigue syndrome, temporomandibular joint dysfunction, migraines, irritable bowel syndrome, multiple chemical sensitivity, anxiety, and depression. All these sociodemographic variables were collected using a self-administered survey.

The following tools were used in this study:

1. Pain: we used a visual analog scale (VAS) to assess self-reported pain intensity, ensuring maximum reproducibility among different observers [32]. Furthermore, we used an analog pressure FPK 20 algometer (Wagner algometer, Force Dial FDK 20; Wagner Instruments) to evaluate pain among individuals diagnosed with FM. This instrument allows the assessment of a patient’s central sensitivity to pain. Measurements focused on tender points in the occipital and upper trapezius regions bilaterally [33-35].
2. Subjective intensity of effort was assessed using the Borg Category-Ratio Scale (CR-10), which quantifies the subjective intensity of effort experienced during physical exercise or functional testing. Participants were asked to rate their exertion immediately after each test or exercise session on a 0 to 10 scale, where 0 indicates “no exertion at all” and 10 represents “maximal exertion” [36].
3. Neck Disability Index (NDI): the validated Spanish version of this questionnaire was used to assess pain and neck-related disability [37].
4. Fear of movement: the Tampa Scale for Kinesiophobia (TSK) was used to assess the fear of movement [38].
5. Exercise adherence: we used the Exercise Adherence Rating Scale, which is a validated questionnaire with 2 sections, one assessing exercise performance and another evaluating frequency, motivation, and consistency [39].
6. Impact of FM: the Fibromyalgia Impact Questionnaire (FIQ) was used to assess the impact of FM on health-related quality of life [40-42].
7. Quality of life: we used EQ-5D (EuroQol 5-Dimensions) questionnaire, which is a tool that allows the evaluation of a patient’s overall quality of life in primary care settings [43-45].
8. Symptoms of central sensitization: we used the validated Spanish version of the Central Sensitization Inventory (CSI). This questionnaire assesses symptoms using a scale of 0 (“never”) to 4 (“always”). The total score ranges from 0 to 100. A score above 40 indicates the presence of central sensitization [46].
9. ROM: cervical flexion, extension, lateral flexion, and rotation were assessed using a goniometer [47]. This instrument is used to evaluate one’s degree of joint mobility, thereby facilitating the determination of an individual’s restrictions.
10. Time Up Go test: the test consisted of measuring the time it took participants to get up from a chair with a height of 46 cm, walking 3 meters, turning around a cone, and sitting down again. This test was performed to assess physical performance, gait, and dynamic balance [48,49].

### Procedure

The intervention was conducted over a period of 1 month. G1 and G2 participated in 2 sessions per week. G1 performed 20 minutes of cervical mobility and strengthening exercises (Multimedia Appendix 1), followed by 10 minutes of immersive VR therapy. G2 completed 30 minutes of cervical mobility and strengthening exercises. The CG did not perform any cervical mobility or strengthening exercises nor participate in immersive VR therapy. The immersive VR therapy was delivered using Meta Quest 2000 headsets, using the game “Interkosmos 2000.” In the game, participants assumed the role of a spacecraft pilot navigating through a series of rings while avoiding meteoroids by performing neck movements according to the game’s instructions. The difficulty level was adjusted as follows: the first 3 sessions were carried out in “easy” mode, allowing a maximum cervical mobility of 30°. The subsequent 3 sessions were conducted in “medium” mode, allowing up to 60° of cervical mobility. The final 2 sessions were performed in “hard” mode, which not only increased the spacecraft’s speed but also enabled a full cervical range of motion. All groups underwent assessments of cervical ROM including flexion, extension, right and left lateral inclination, and right and left rotation. In addition, pain intensity in the upper trapezius (bilaterally) and in the right and left occipital regions was quantified using an algometer. Evaluations were conducted at 4 time points: baseline (preintervention), immediately after the intervention, 15 days postintervention, and 1 month postintervention. All follow-up assessments were performed at the association’s premises. After each session, participants from G1 and G2 reported their perceived levels of cervical pain and fatigue. All outcome measures were collected by a research assistant blinded to the study objectives and group allocations.

Evaluations were conducted at 4 time points: baseline (preintervention), immediately postintervention, 15 days postintervention, and 1-month postintervention. All follow-up assessments were conducted at the association’s headquarters. After each session, participants in G1 and G2 reported perceived levels of cervical pain and fatigue.

All outcome measures were collected by a research assistant who was blinded to both the study’s objectives and the participants’ group assignments.

### Statistical Analysis

Statistical analyses were performed using SPSS 29.0 (IBM Corp). The normality of quantitative variables was assessed with the Kolmogorov-Smirnov test. Depending on distribution, data were described using means and SDs or medians, IQRs, and ranges. Qualitative variables were expressed as percentages and absolute values. Baseline comparisons between the groups were conducted using chi-squared tests or ANOVA with Bonferroni post hoc analysis. Temporal changes in outcomes were analyzed with paired tests, and intergroup differences were analyzed using ANOVA, Bonferroni post hoc, or Kruskal-Wallis tests, as appropriate. Correlations between quantitative variables were assessed using the Pearson or Spearman tests, and associations between qualitative variables were assessed using chi-squared tests. Statistical significance was set at *P*<.05.

### Ethical Considerations

This study received approval from the institutional review board of Hospital Clínico San Carlos (23‐458 EC) and was conducted in accordance with the Spanish legislation, including Law 41/2002 on patient autonomy and Organic Law 3/2018 on data protection and digital rights. These laws prohibit the processing of sensitive personal data such as racial or ethnic origin, political or religious beliefs, biometric identifiers, health information, and sexual orientation. The study also adhered to the ethical principles outlined in the World Medical Association’s Declaration of Helsinki 2014 [50]. All procedures conducted in this study complied with applicable ethical standards. Informed consent was obtained from all participants prior to their inclusion in the study. Participant privacy and confidentiality were strictly protected, and all data were handled and stored in accordance with relevant data protection regulations. No personally identifiable information was collected or disclosed. Participants did not receive any financial or material compensation for their participation.

## Results

### Participant and Baseline Characteristics

**Figure 1.**
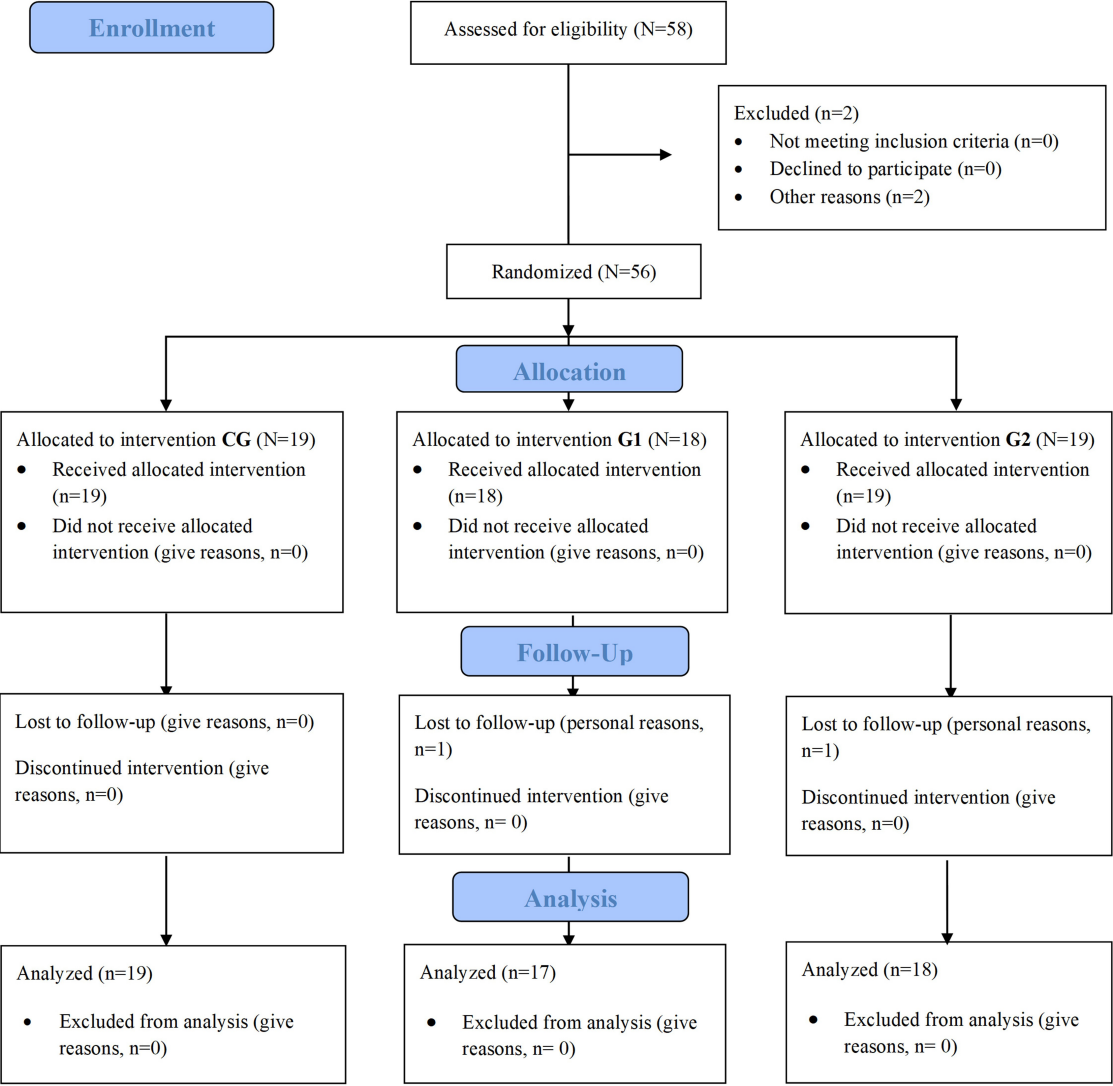
CONSORT (Consolidated Standards of Reporting Trials) flow diagram. CG: control group.

The final sample included 54 women with a mean age of 54.26 (SD 7.7) years. Participants were distributed among the groups by computer-generated random number sequence (Figure 1).

Among the participants, 33 (61%) presented chronic fatigue syndrome, 37 (68%) had temporomandibular joint dysfunction, and 16 (33%) reported restless legs syndrome. Regarding the psychological comorbidities, 33 (61%) participants presented anxiety and 13 (24%) depression. Baseline comparisons revealed no statistically significant differences among groups in age, BMI, or other demographic variables. However, subjective measures showed group-level differences in several baseline scales, including FIQ, EQ-5D, CSI, NDI, and Borg scale. Almost all variables at baseline start from similar values, although G1 has a value of 1 point lower on the VAS scale, indicating that they have less pain at the outset. However, in variables such as algometry, the averages reflect similar values compared to the other 2 groups (Multimedia Appendix 2).

### Postintervention Outcomes

Table 1 shows the differences in the mean values measured before therapy and immediately after its end. We found significant differences in all groups that received intervention in FIQ, CSI, NDI, right and left trapezius, occipital algometer, and in all movements in which ROM was evaluated. For the right and left trapezius and occipital muscles, algometric measurements were obtained, and ROM was assessed across all evaluated movements. About the treatment adherence variable, G1 obtained an average of 94 points out of 100, compared to G2, whose post-treatment average was 74.72. The summary of the comparison between baseline measures and measures immediately after intervention (intragroup comparisons). Shown are the differences in the mean values between both time points (Multimedia Appendix 3).

**Table 1.** Intragroup (G1, G2, and CG^a^) analysis of variables measured using questionnaires between baseline and immediately after intervention.

Value and intervention group		Differences of mean values (95% CI)	*P* value
FIQ^b^ (1-100)			
Whole series		5.4 (3.2 to 7.5)	<.001
G1		9 (4.6 to 13.3)	<.001
G2		8.1 (5.5 to 10.6)	<.001
CG		0.36 (−3.2 to 2.51)	.80
EQ-5D^c^ (–0.59 to 1)			
Whole series		4.1 (2.6 to 5.6)	<.001
G1		3.1 (0.6 to 5.6)	.01
G2		7.7 (5.6 to 9.8)	<.001
CG		−1.7 (−4.4 to 1)	.20
TSK^d^ (17-37)			
Whole series		3.2 (0.8 to 5.6)	.01
G1		1.2 (−5.2 to 7.6)	.70
G2		6.2 (4.7 to 9.8)	<.001
CG		−2.04 (−5.9 to 1.86)	.30
CSI^e^ (0-100)			
Whole series		4.2 (2.4 to 6)	<.001
G1		6 (3.5 to 8.5)	<.001
G2		7.2 (4.7 to 9.8)	<.001
CG		0.32 (−3.5 to 2.9)	.80
NDI^f^ (0-50)			
Whole series		4.5 (3.1 to 5.9)	<.001
G1		5.35 (3.2 to 7.5)	<.001
G2		7.4 (5.1 to 9.7)	<.001
CG		−0.95 (−2.9 to 1.05)	.33

a CG: control group.

b FIQ: Fibromyalgia Impact Questionnaire.

c EQ-5D: EuroQol 5-Dimensions.

d TSK: Tampa Scale for Kinesiophobia.

e CSI: Central Sensitization Inventory.

f NDI: Neck Disability Index Questionnaire.

### Follow-Up at 1 Month

Summary of the comparison between baseline and 1-month post-intervention scores for FIQ, EQ-5D, TSK, CSI, and NDI (intragroup comparisons). Shown are the differences in the mean values between both time points (Table 2). The differences in the measurement times of the other variables can be observed in Multimedia Appendix 4.

**Table 2.** Intragroup (G1, G2, and CG^a^) analysis of variables measured using questionnaires between baseline and 1 month after intervention.

Value and intervention group		Differences of mean values (95% CI)	*P* value
FIQ^b^			
Whole series		4.4 (2.5 to 76.3)	<.001
G1		10.71 (6.6 to 14.8)	<.001
G2		2.71 (0.55 to 4.87)	.02
CG		0.33 (–2.08 to 1.42)	.70
EQ-5D^c^			
Whole series		4.7 (2.6 to 5.7)	<.001
G1		7.7 (5.46 to 9.97)	<.001
G2		4.17 (1.7 to 6.63)	.002
CG		1.07 (–3.87 to 1.73)	.43
TSK^d^			
Whole series		3.3 (1.4 to 5.1)	.001
G1		8.41 (5.25 to 11.58)	<.001
G2		2.1 (0.06 to 4.13)	.04
CG		0.2 (–2.87 to 3.28)	.89
CSI^e^			
Whole series		3.9 (2.18 to 5.8)	<.001
G1		9.78 (7.35 to 12.22)	<.001
G2		2.94 (1.25 to 4.64)	.02
CG		0.26 (–2.76 to 3.3)	.85
NDI^f^			
Whole series		4.03 (2.5 to 6.31)	<.001
G1		10.65 (8.35 to 12.94)	<.001
G2		4.22 (2.39 to 6.06)	<.001
CG		2.05 (–0.33 to 4.43)	.08

a CG: control group.

b FIQ: Fibromyalgia Impact Questionnaire.

c EQ-5D: EuroQol 5-Dimensions.

d TSK: Tampa Scale for Kinesiophobia.

e CSI: Central Sensitization Inventory.

f NDI: Neck Disability Index Questionnaire.

After confirming the effect of both interventions, we compared the intervention groups to see whether VR had a significant influence on the effect of the therapy. Table 3 shows that the ANOVA between groups was significant for FIQ, CSI, and NDI at both postintervention and 1-month follow-up (*P*<.001). Immediately after treatment, both intervention groups (G1 and G2) improved significantly compared with the CG, with no differences between G1 and G2. At 1 month, G1 maintained superior outcomes across all measures, showing significantly better scores than both GC and G2 (*P*<.001), while G2 no longer differed from GC for FIQ and CSI. For NDI, all pairwise comparisons were significant (*P*<.001), confirming that both interventions reduce neck disability, with the VR-enhanced program providing the greatest and most sustained benefit.

**Table 3.** Summary of the comparison between the groups for the change in FIQ^a^, CSI^b^, and NDI^c^, both immediately after intervention and 1 month after.

Variable and intervention group		*P* value for ANOVA	Mean difference between time points (95% CI)	*P* value
FIQ				
Immediate		<.001		
CG^d^ vs G1			9.31 (14.7 to 3.8)	<.001
CG vs G2			8.4 (13.84 to 3.06)	.001
G2 vs G1			0.85 (6.39 to −4.68)	>.99
1 month		<.001		
CG vs G1			10.38 (5.8 to 14.9)	<.001
CG vs G2			2.38 (–2.12 to 6.9)	.59
G2 vs G1			7.99 (3.35 to 12.63)	<.001
CSI				
Immediate		<.001		
CG vs G1			6.31 (10.96 to 1.66)	.04
CG vs G2			7.53 (12.12 to 2.95)	<.001
G2 vs G1			–1.22 (3.4 to −5.93)	.51
1 month		<.001		
CG vs G1			10.04 (14.16 to 5.93)	<.001
CG vs G2			3.20 (7.26 to −0.84)	.16
G2 vs G1			6.84 (11.01 to 2.67)	.001
NDI				
Immediate		<.001		
CG vs G1			4.40 (7.95 to 0.85)	.01
CG vs G2			6.44 (9.93 to 2.94)	<.001
G2 vs G1			–2.03 (1.55 to −5.62)	.50
1 month		<.001		
CG vs G1			12.69 (16.33 to 9.06)	<.001
CG vs G2			6.27 (9.85 to 2.69)	<.001
G2 vs G1			6.42 (10.10 to 2.74)	<.001

a FIQ: Fibromyalgia Impact Questionnaire.

b CSI: Central Sensitization Inventory.

c NDI: Neck Disability Index Questionnaire.

d CG: control group.

## Discussion

### Principal Findings

This study evaluated the effectiveness of VR as an adjunctive therapy for cervical pain in women with FM. The main findings revealed that combining VR with mobility and strengthening exercises produced significant improvements in pain perception, ROM, and functional performance compared with physical therapy alone. These benefits were also sustained at 1-month follow-up, emphasizing the potential of immersive VR interventions to enhance therapeutic outcomes in individuals with FM.

In the intergroup analysis, the G1 consistently outperformed both G2 and CG in those outcomes most closely related to global disease impact and NDI. Specifically, G1 showed significantly greater and more sustained improvements in FIQ and CSI scores than both CG and G2, whereas improvements in G2 tended to converge toward control values at 1-month follow-up. Similarly, NDI scores decreased in both intervention groups immediately after treatment, but at follow-up, a clear gradient GC>G2>G1 was observed, indicating the largest and most persistent reduction in neck-related disability in the combined intervention. These effects were paralleled by a recurrent pattern of superior gains in cervical ROM and higher-pressure pain thresholds in the trapezius and occipital in G1, while the Time Up Go test improved in both intervention groups compared with CG and TSK decreased more markedly in G1 at 1 month. By contrast, the VAS and Borg scale showed only limited discrimination between the groups, suggesting that the added value of VR was more evident in multidimensional impact and sensitization-related and functional domains than in isolated pain ratings.

This intergroup pattern is consistent with previous evidence in FM and chronic neck pain, indicating that VR, particularly when used as an adjunct to active exercise, preferentially enhances global impact, disability, and pain-modulation outcomes. In patients with FM, a study reported that fully immersive VR combined with aerobic and pilates training produced greater improvements in pain, kinesiophobia, fatigue, physical activity levels, and mental quality of life than exercise alone, while both groups improved in FM impact, supporting an adjunctive role of immersive VR in comprehensive rehabilitation programs [8]. Other studies showed that, in individuals with chronic neck pain, VR-based cervical training led to larger gains in pressure pain thresholds at the upper cervical levels and greater reductions in functional limitation compared with motor control exercises, despite the absence of between-group differences in pain intensity and quality of life, which parallels our finding of more robust intergroup differences in pressure pain thresholds, ROM, and disability than in VAS score [51]. Furthermore, a randomized crossover trial in women with FM found that VR increased cold pain thresholds and tolerance in both FM patients and pain-free controls, whereas effects on pain intensity were limited, reinforcing the notion that VR may primarily modulate pain processing rather than consistently decreasing reported pain intensity [52]. Taken together, these converging results suggest that combining immersive VR with targeted cervical exercise may primarily potentiate mechanisms related to central sensitization, motor control, and the global impact of FM rather than merely amplifying short-term analgesic effects.

VR has emerged as a promising nonpharmacological intervention for the symptom management of FM. Its immersive and interactive features provide cognitive distraction, emotional engagement, and motor stimulation, which together enhance adherence and reduce pain intensity. Previous studies have demonstrated that VR can improve mood, motivation, and functional capacity when applied alongside traditional rehabilitation or psychological therapies. This approach not only benefits patients with FM but may also be applicable to other chronic pain conditions [49,50,53].

The use of VR has been demonstrated to significantly influence pain relief, motor function, and joint mobility among patients with a range of chronic pathologies [53]. The primary applications of VR in health care include the management of pain and anxiety as well as the enhancement of patient motivation [47,54,55].

While it is not possible to make direct comparisons between this study and research conducted by other authors, the described implementation of an 8-session intervention resulted in statistically significant outcomes for the variables under investigation. This suggests VR is effective when combined with active exercise therapy.

The comparative analysis concerning VR and physical exercise indicated that there was minimal divergence in the measured variables. In a separate study [56], 44 patients underwent an intervention for 4 weeks, with 2 sessions per week. The intervention was divided into 2 groups: G1 (whose members performed only cervical mobility exercises) and G2 (whose members conducted a treatment based solely on VR exercises). The findings indicated that there were no statistically significant differences between the 2 groups across the different variables, including pain, ROM, neck disability, incapacitating pain, and anxiety. However, in this study, the combination of VR with cervical mobility exercises yielded a significantly better outcome. Furthermore, these improvements were maintained over time (1 month) to a greater degree in the group subjected to both therapies combined when compared to the group that performed only mobility exercises. It is important to highlight that the study with which this investigation was compared implemented its intervention in a population without FM. Consequently, the observed discrepancy in outcomes could be due to this factor. A similar study was conducted [51], with an equivalent number of sessions implemented. The outcomes observed in this research were analogous to those obtained in our research, as the investigators combined active therapy and VR exercises for patients with FM [55]. However, the FM cohort in that report comprised only 20 patients. In addition, the active component focused on aerobic training and Pilates instead of the cervical exercises used in our research.

Another study assessed the efficacy of VR for patients with chronic neck pain versus a CG that performed motor control exercises. In this case, the study spanned a period of 6 weeks, comprising 3 weekly sessions. Their findings revealed that the utilization of VR was advantageous when measuring pain due to pressure with an algometer at C1-C2 and C5-C6, and it was also beneficial when considering functional limitations. However, no significant differences were observed between the groups with respect to pain intensity, muscle performance, or quality of life (36-Item Short Form Health Survey). It is evident that the results of this study deviate from those previously observed. However, it should be noted that the mentioned study did not include the population with FM, and no group underwent combined VR and active therapy [51].

Following a thorough review of the scientific literature, we found no studies addressing the use of VR in the cervical region using this protocol. Other studies have applied VR in FM use, games, and global exercises, focusing on observing aspects such as anaerobic capacity, balance, or fatigue [7,54,55]. In this study, the focus was pain in the cervical region and other more global variables such as fatigue, VAS, and FIQ. Therefore, our study offers a novel contribution to this field of research by addressing this gap in the existing literature. Given that patients with FM experience cervical pain, headaches, and tender points in the occipital regions, it is imperative to reduce the associated symptoms.

The limitations of this study lie in the fact that the results are not representative of a heterogeneous population with FM, since the cohort of participants in this research consisted solely of women. Due to the challenging nature of the disease, it was not possible to consider medication used by participants, which may have influenced the results. Finally, the study did not include a group that used only VR, so we cannot conclude that the results are attributable to the VR component or its combination with exercise.

This approach not only benefits patients with FM but may also be applicable to other chronic pain conditions [49,50,53].

### Conclusions

Virtual reality, when added to cervical mobility and strengthening exercises, produced greater and more sustained improvements in disease impact, neck disability, central sensitization, kinesiophobia, and functional outcomes in women with FM than exercise alone or no intervention. The improvements were maintained for 1 month in the G1 group in the variables evaluated. These findings support VR as an effective adjunct to active therapy, with potential benefits for symptom reduction and warrant further research on long-term effects, cost-effectiveness, and applicability to broader chronic pain populations.

## Supplementary material

10.2196/81158 Multimedia Appendix 1 Recommended exercises.

10.2196/81158 Multimedia Appendix 2 Baseline measurements of pain and functional capacity (mean and SD).

10.2196/81158 Multimedia Appendix 3 Intragroup analysis of baseline and immediately after intervention.

10.2196/81158 Multimedia Appendix 4 Intragroup analysis of baseline and 1 month after intervention.

10.2196/81158 Checklist 1 CONSORT-Harms-2022 checklist.

## Abbreviations

CG: control group
CONSORT: Consolidated Standards of Reporting Trials
CONSORT-Harms: Consolidated Standards of Reporting Trials—Harms Extension
CSI: Central Sensitization Inventory
EQ-5D: EuroQol Dimensions
FIQ: Fibromyalgia Impact Questionnaire
FM: fibromyalgia
NDI: Neck Disability Index Questionnaire
ROM: range of motion
TSK: Tampa Scale for Kinesiophobia
VAS: visual analog scale
VR: virtual reality
