# Supplementary material for: Therapeutic Use of Virtual Reality for Patients With Fibromyalgia and Chronic Neck Pain: Randomized Controlled Trial
Source: JMIR Rehabil Assist Technol. 2026 Jan 23;13:e81158. doi: 10.2196/81158 (PMC12829586; doi:10.2196/81158)
Supplement: Multimedia Appendix 1 [file rehab-v13-e81158-s001.docx]

**Multimedia Appendix 1**. Recommended exercises.

| **Cervical stretching** | Posterior Extensor Muscles | **Cervical Strengthening** | Isometric exercises (neck flexion-extension, lateral flexion, and rotation) | **Cervical Mobility** | Circular movements |
| --- | --- | --- | --- | --- | --- |
|  | Lateral flexor muscles |  | Shoulder flexion-extension |  | Flexion-extension |
|  | Posterior scalene and levator scapulae |  | Cervicobrachial mobility |  | Rotation |
|  | Anterior and middle scalene, and sternocleidomastoid |  | Rebound exercises |  | Lateral flexion |
