# Supplementary material for: Therapeutic Use of Virtual Reality for Patients With Fibromyalgia and Chronic Neck Pain: Randomized Controlled Trial
Source: JMIR Rehabil Assist Technol. 2026 Jan 23;13:e81158. doi: 10.2196/81158 (PMC12829586; doi:10.2196/81158)
Supplement: Multimedia Appendix 2 [file rehab-v13-e81158-s002.docx]

Multimedia Appendix 2. Baseline measurements of pain and functional capacity (mean and standard deviations).

| **Variable** | **SAMPLE**  **(n=54)** | **G1** | **G2** | **CG** | ***P* -value** |
| --- | --- | --- | --- | --- | --- |
|  |  | **(n=17)** | **(n=18)** | **(n=19)** |  |
|  |  |  |  |  |  |
| **FIQ** | 69.8±10.2 | 64.6±14.2 | 71.7±6.8 | 72.8±6.9 | .030 |
| **EQ-5D** | 72.5±95 | 63.1±9.0 | 76.3±4.4 | 77.5±7.3 | <.010 |
| **TSK** | 63.35±16,7 | 61.1±12.5 | 59.9±20.3 | 68.6±15.7 | .200 |
| **CSI** | 69.69±9.1 | 64.0±11.1 | 72.2±8.4 | 72.4±4.8 | <.010 |
| **NDI** | 68.25±12.39 | 58.4±13.4 | 75,8±9.2 | 69.9±7.8 | <.010 |
| **TUG** | 9.8±2.4 | 9.7±2.2 | 10.3±3.0 | 9.6±2.2 | .600 |
| **VAS** | 6.7±1.8 | 6.1±2.1 | 7.0±1.4 | 7.1±1.8 | .200 |
| **BORG SCALE** | 5.9±2.26 | 4.7±2.2 | 6.7±2.2 | 6.3±2.0 | .010 |
| **LEFT TRAPEZIUS ALGOMETER** | 3.3±1,7 | 3.5±1.7 | 3.0±2.1 | 3.4±1.3 | .660 |
| **RIGHT TRAPEZIUS ALGOMETER** | 3.3±1.5 | 3.8±1.1 | 2.8±1.8 | 3.5±1.4 | .150 |
| **LEFT OCCIPITAL ALGOMETER** | 2.9±1.38 | 2.9±1.5 | 2.6±1.7 | 3.3±1.0 | .380 |
| **RIGHT OCCIPITAL ALGOMETER** | 2.8±1.5 | 2.8±1.4 | 2.3±1.7 | 3.6±1.1 | .050 |
| **FLEXION ROM** | 37.67±10.87 | 35.0±10.1 | 38.2±11.5 | 39.6±11.0 | .450 |
| **EXTENSION ROM** | 31.65±7.9 | 33.6±9.3 | 31.4±6.0 | 30.2±8.3 | .430 |
| **RIGHT LATERAL FLEXION ROM** | 31.52±7.2 | 31.4±5.4 | 31.3±7.7 | 31.8±8.6 | .970 |
| **LEFT LATERAL FLEXION ROM** | 30.8± 8.9 | 28.2±7.7 | 31.8±10.98 | 50.89±10.2 | .350 |
| **RIGHT ROTATION ROM** | 51.3±9.6 | 54.06±8.9 | 49±9.4 | 50.9±10.2 | .300 |
| **LEFT ROTATION ROM** | 48.31±9.5 | 47.6±8.6 | 48.9±7.0 | 48.4±12.3 | .900 |

FIQ (Fibromyalgia Impact Questionnaire); EQ-5D (EuroQol 5-Dimensions); TSK (Tampa Scale for Kinesiophobia); CSI (Central Sensitization Inventory); NDI (Neck Disability Index Questionnaire); VAS (visual analogic scale).
