# Supplementary material for: Therapeutic Use of Virtual Reality for Patients With Fibromyalgia and Chronic Neck Pain: Randomized Controlled Trial
Source: JMIR Rehabil Assist Technol. 2026 Jan 23;13:e81158. doi: 10.2196/81158 (PMC12829586; doi:10.2196/81158)
Supplement: Multimedia Appendix 3 [file rehab-v13-e81158-s003.docx]

**Multimedia Appendix 3.** Intra-group analysis of baseline and immediately after intervention.

| **Value** | **Intervention group** | **Differences of mean values (95% CI)** | ***p*-value** |
| --- | --- | --- | --- |
| **VAS** | Whole series | 0.02 (-0.4-0.37) | .920 |
|  | G1 | 0.2 (-0.9-0.37) | .600 |
|  | G2 | 0.4 (-1.2-0.7) | .698 |
|  | CG | -0.2 (-0.7-0.3) | .410 |
| **Right trapezius algometer** | Whole series | 0.55 (0.28-0.983) | < .001 |
|  | G1 | 0.8 (0.4-1.1) | < .001 |
|  | G2 | 1.2 (1.6-6.2) | < .001 |
|  | CG | -0.24 (-0.7-0.21)) | .270 |
| **Left trapezius algometer** | Whole series | 0.5 (0.23-0.77) | <.001 |
|  | G1 | 0.64 (0.03-1.26) | .040 |
|  | G2 | 0.9 (0.4-1.4) | <.001 |
|  | CG | 0.02 (-0.26-0.22) | .850 |
| **Right occipital algometer** | Whole series | 0.59 (0.22-0.95) | .002 |
|  | G1 | 1.2 (0.78-1.54) | < .001 |
|  | G2 | 1.2 (0.66-1.7) | < .001 |
|  | CG | -0.46 (-1.1-0.2) | .150 |
| **Left occipital algometer** | Whole series | 0.49 (0.23-0.75) | < .001 |
|  | G1 | 0.95 (0.52-1.39) | < .001 |
|  | G2 | 0.64 (0.14-1.24) | .016 |
|  | CG | -0.05 (-0.29-0.4) | .740 |
| **TUG** | Whole series | 0.7 (0.3-1.1) | .001 |
|  | G1 | 0.98 (0.3-1.7) | .008 |
|  | G2 | 1.4 (0.4-2.4) | .009 |
|  | CG | 0.22 (-0.45-0.06) | .055 |
| **Borg Scale** | Whole series | 0.04 (-0.4-0.4) | .870 |
|  | G1 | 0.23 (-0.65-1.1) | .579 |
|  | G2 | 0.17 (-0.7-1.1) | .270 |
|  | CG | 0.48 (-1.1-0.16) | .130 |
| **Cervical flexion ROM** | Whole series | 6.7 (5-9.4) | <.001 |
|  | G1 | 8.9 (4.2-13.6) | .001 |
|  | G2 | 13.2 (10-16.36) | <.001 |
|  | CG | -1.48 (-5.1-2.2) | .410 |
| **Cervical extension ROM** | Whole series | 6.9 (4.9-9.1) | <.001 |
|  | G1 | 11 (7.2-14.8) | <.001 |
|  | G2 | 9.7 (6.4-13) | <.001 |
|  | CG | 0.8 (-1.4-3) | .470 |
| **Right lateral flexion ROM** | Whole series | 3.9 (2.1-5.7) | <.001 |
|  | G1 | 6.7 (2.7-10.7) | 013 |
|  | G2 | 5.2 (2.7-7.8) | < .001 |
|  | CG | 0.1 (-2.7-2.5) | .930 |
| **Left lateral flexion ROM** | Whole series | 4.6 (2.6-6.7) | < .001 |
|  | G1 | 6.1 (1.5-10.8) | .003 |
|  | G2 | 7.7 (4.1-11.3) | < .001 |
|  | CG | 0.42 (-2.3-1.4) | .640 |
| **Right rotation ROM** | Whole series | 7.6 (5.4-9.8) | < .001 |
|  | G1 | 9.8 (6.2-13.5) | < .001 |
|  | G2 | 12.6 (8.9-16.3) | < .001 |
|  | CG | 0.84 (-2.9-1.2) | 0.410 |
| **Left rotation ROM** | Whole series | 9.8 (7.1-12.5) | < .001 |
|  | G1 | 14.2 (8.7-19.6) | < .001 |
|  | G2 | 14.8 (11.2-18.4) | < .001 |
|  | CG | 1.1 (-1.2-0.8) | .250 |

. VAS: Visual Analogue Scale; TUG: Time Up Go and ROM: Range of Movement
