# Supplementary material for: Therapeutic Use of Virtual Reality for Patients With Fibromyalgia and Chronic Neck Pain: Randomized Controlled Trial
Source: JMIR Rehabil Assist Technol. 2026 Jan 23;13:e81158. doi: 10.2196/81158 (PMC12829586; doi:10.2196/81158)
Supplement: Multimedia Appendix 4 [file rehab-v13-e81158-s004.docx]

**Multimedia Appendix 4.** Intra-group analysis of baseline and 1 month after intervention.

| **Value** | **Intervention group** | **Differences of mean values (95% CI)** | ***p*-value** |
| --- | --- | --- | --- |
| **VAS** | Whole series | 0.22 (-0.12-0.58) | .190 |
|  | G1 | 0.88 (1.48-2.83) | .007 |
|  | G2 | 0.05 (-0.522-0.63) | .826 |
|  | CG | 0.11 (-0.54-0.77) | .700 |
| **Right trapezius algometer** | Whole series | 0.41 (0.16-0.65) | .002 |
|  | G1 | 1.02 (0.68-1.37) | <.001 |
|  | G2 | 10.56 (0.24-0.87) | .001 |
|  | CG | 0.28 (-0.24-0.7) | .170 |
| **Left trapezius algometer** | Whole series | 0.37 (0.12-0.63) | .005 |
|  | G1 | 1.08 (0.61-1.55) | <.004 |
|  | G2 | 0.27 (-0.17-0.71) | .258 |
|  | CG | 0.13 (-0.15-0.41) | .350 |
| **Right occipital algometer** | Whole series | 0.48 (0.1-0.87) | .013 |
|  | G1 | 1.58 (1.18-1.97) | <.001 |
|  | G2 | 0.65 ((0.19-1.1) | .008 |
|  | CG | 0.64 (-0.02-1.3) | .060 |
| **Left occipital algometer** | Whole series | 0.42 (0.13-0.72) | .006 |
|  | G1 | 1.36 (0.93-1.79) | <.001 |
|  | G2 | 0.27 (-0.17-0.71) | .211 |
|  | CG | -0.05 (-0.29-0.4) | .150 |
| **TUG** | Whole series | 0.85 (0.46-1.24) | <.001 |
|  | G1 | 1.26 (1-2.26) | < .001 |
|  | G2 | 1.1 (0.3-1.97) | .011 |
|  | CG | 0.11 (-0.17-0.4) | .400 |
| **Borg Scale** | Whole series | 0 (-0.36-0.36) | 1.000 |
|  | G1 | 0.47 (-0.36-1.31) | .250 |
|  | G2 | 0.05 (-0.52-0.63)) | .840 |
|  | CG | 0.47 (-0.04-0.992) | .070 |
| **Cervical flexion ROM** | Whole series | 6.37 (4.1-8.7) | < .001 |
|  | G1 | 14.06 (10.09-18.02) | <.001 |
|  | G2 | 5.89 (3.1-8.6) | < .001 |
|  | CG | 0.28 (-0.11-0.68) | .960 |
| **Cervical extension ROM** | Whole series | 6.4 (4.1-8.7) | < .001 |
|  | G1 | 14.3 (10.34-18.25) | < .001 |
|  | G2 | 5.78 (2.78-8.78) | .001 |
|  | CG | 0 (-1.9-1.9) | 1.000 |
| **Right lateral flexion ROM** | Whole series | 4.1 (2.14-6.12) | < .001 |
|  | G1 | 10.06 (6.57-13.54) | < .001 |
|  | G2 | 2.89 (0.79-5) | .010 |
|  | CG | 0 (-3.25-3.25) | 1.000 |
| **Left lateral flexion ROM** | Whole series | 3.89 (2.25-5.52) | < .001 |
|  | G1 | 8.9 (5.67-12.21) | < .001 |
|  | G2 | 2.1 (-0.19-4.31) | .071 |
|  | CG | 1.1 (-0.77-2.98) | .230 |
| **Right rotation ROM** | Whole series | 6.1 (3.7-8.4) | < .001 |
|  | G1 | 13.59 (10.42-16.75) | < .001 |
|  | G2 | 7.72 (4.44-11-01) | < .001 |
|  | CG | 2.16 (-0.58-3.8) | .100 |
| **Left rotation ROM** | Whole series | 8.26 (5.36-11.16) | < .001 |
|  | G1 | 17.29 (12.38-22.21) | < .001 |
|  | G2 | 9.33 (5-13.68) | < .001 |
|  | CG | 0.84 (-0.87-2.55) | .310 |

VAS: Visual Analogue Scale; TUG: Time Up Go and ROM: Rate of Movement.
